# Clinical, virological and immunological characteristics of people living with HTLV at a Swedish tertiary referral center

**DOI:** 10.1186/s12879-026-14761-x

**Published:** 2026-11-12

**Authors:** Anna-Karin Svensson, Jan Vesterbacka, Sara Gredmark-Russ, Martin Sundqvist, Martin Paucar, Christer Lidman, Piotr Nowak

**Affiliations:** 1 Department of Infectious Diseases, Karolinska University Hospital, Stockholm, Sweden; 2 Department of Medicine Huddinge, Center for Infectious Medicine, Karolinska Institutet, Stockholm, Sweden; 3 School of Medical Sciences, University of Örebro, Örebro, Sweden; 4 Department of Clinical Neuroscience, Karolinska University Hospital, Stockholm, Sweden; 5 Department of Clinical Neuroscience, Karolinska Institutet, Stockholm, Sweden

**Keywords:** HTLV, ATLL, HAM/TSP, HTLV in Sweden, MTCT in Sweden

## Abstract

**Background:**

The prevalence of HTLV in Sweden is presumed to be low, however comprehensive data are lacking. This study describes clinical and laboratory characteristics of patients with HTLV infection followed at the outpatient clinic of Karolinska University Hospital in Stockholm, Sweden.

**Methods:**

We performed a retrospective analysis of all HTLV positive patients followed at our clinic between January 2013 and May 2024. Demographics, mode of transmission, HTLV type, proviral load (PVL), co-infections, diagnosis of HTLV-associated myelopathy/tropical spastic paraparesis (HAM/TSP) or adult T-cell leukemia/lymphoma (ATLL), and immunological markers were extracted from medical records.

**Results:**

We included 102 patients (82% HTLV-1, 18% HTLV-2). The median age was 56 years (IQR 42–62; range 23–81) and 54% were women. Twenty-nine percent of the patients were born in Sweden, while the rest had other countries of origin (33% Middel East; 16% South America; 11% Africa, and 5% the Caribbean). HTLV infection was identified by contact tracing (35%), blood donor or IVF screening (31%), and symptom-based testing (7%). Mother-to-child transmission was considered a possible route of infection in four Swedish born individuals. Ten individuals (12% of people living with HTLV-1) had HAM/TSP, and two were diagnosed with ATLL. Patients with HAM/TSP had higher median PVL than asymptomatic carriers. The proportion of regulatory T cells were higher in symptomatic individuals and was positively correlated with PVL.

**Conclusions:**

This study provides the first comprehensive characterization of a cohort of people living with HTLV in Sweden. Despite the low national prevalence, we observed a notable proportion of symptomatic cases, including HAM/TSP and ATLL. We also observed four cases of MTCT in individuals born in Sweden.

## Background

Human T Cell Lymphotropic Virus (HTLV) is a retrovirus estimated to affect 5–10 million people globally [1]. Discovered in 1979 in a patient with T cell leukemia, it was the first oncogenic human virus ever isolated [2]. HTLV is endemic in specific regions, including southern Japan, South America, the Middle East, the Caribbean, parts of Africa, and Melanesia [1]. While the vast majority of HTLV-infected individuals remain asymptomatic, approximately 5% develop serious clinical disease associated with the virus. HTLV-1 is the causative agent of adult T cell leukemia/lymphoma (ATLL)-an aggressive and difficult-to-treat hematologic malignancy. In addition, HTLV-1 is linked to HTLV-1-associated myelopathy/tropical spastic paraparesis (HAM/TSP), a chronic neuroinflammatory disorder of the spinal cord typical presenting with progressive lower limb weakness, spasticity, and bladder dysfunction, which may ultimately lead to loss of ambulation [3]. Moreover, HTLV-1 has been connected to a range of inflammatory conditions including uveitis, dermatitis, Sjögren’s syndrome, and arthritis [4]. In contrast, HTLV-2 has not yet been definitively linked to any specific clinical syndrome [5]. The reasons behind the diverse clinical spectrum and the association with inflammatory disease remain incompletely understood.

The prevalence of HTLV in Sweden is not well studied but is estimated to be low (<0.005%) [6]. Several prevalence studies conducted in Sweden between the 1990s and 2007 indicated higher rates of HTLV-1 among specific risk groups, such as intravenous drug users (IDU) and individuals originating from HTLV-1 endemic regions [7, 8]. The most recent study, conducted in 2012 among IDU in Stockholm, reported an HTLV-2 prevalence of 3.2% [7]. However, demographic shifts over the past 15 years may have altered the prevalence landscape.

The Infectious Diseases Department at Karolinska University Hospital, Stockholm, Sweden, serves as a tertiary referral center for HTLV positive patients in Stockholm. In this study, we present a comprehensive overview of the clinical and laboratory characteristics of patients living with HTLV infection who are under regular follow-up at our outpatient clinic.

## Material and methods

### Study population

We conducted a descriptive retrospective study of a clinical cohort comprising all patients diagnosed with HTLV infection and referred to our outpatient clinic at the Karolinska University Hospital, Huddinge, Stockholm, Sweden, between January 2013 and May 31, 2024.

The clinic serves as the primary referral center for HTLV care in Stockholm County that populates around 2,4 million inhabitants [9]. The primary aim of the study was to characterize the demographic, clinical and immunological features of the cohort.

### Data collection

Clinical and laboratory data were retrospectively extracted from our medical records and included the following variables: date of HTLV diagnosis, type (HTLV-1 or HTLV-2), demographics (age, sex, year of diagnosis, year of follow up), region of origin, presumed mode of HTLV transmission, selected HTLV associated comorbidities and autoimmune disorders (defined by ICD −10 codes as hypothyroidism, rheumatoid arthritis, vitiligo, injection drug use (IDU), co-infection with HIV, strongyloidiasis infection and psychiatric disorders (schizophrenia, anxiety)) as well as symptoms of arthralgia/backpain.(defined by ICD-10 codes).

### Study groups within the cohort

Presence of symptomatic disease, specifically HTLV-associated myelopathy/tropical spastic paraparesis (HAM/TSP) and adult T-cell leukemia/lymphoma (ATLL) was documented. The diagnosis of HAM/TSP was based on clinical presentation, excluding other neurological diseases and detection of HTLV antibodies in both serum and cerebrospinal fluid [10]. ATLL diagnosis was based on WHO criteria [11]. Clinical subgroups were mutually exclusive. The arthralgia/backpain subgroup included patients with arthralgia and/or backpain symptoms who did not have HAM/TSP, ATLL, rheumatological disease or autoimmune disease. The subgroups were defined prior to the statistical analysis of PVL and immunology characteristics.

### Immunological testing and PVL analysis

As part of routine clinical care, patients underwent immune profiling, including flow cytometric assessment of CD4^+^ and CD8^+^ T-cell frequencies and calculation of the CD4/CD8 ratio. Regulatory T cells were characterized as T-cells with CD4+CD25+CD127- phenotypes. Immunological analyses were performed at the Immunology department at Karolinska University Hospital.

The HTLV-DNA proviral load (PVL) was measured using droplet digital PCR (ddPCR) performed on DNA extracted from buffy coat samples at the Department of Clinical Microbiology, Örebro University Hospital, Sweden as previously described [12]. The assay quantifies the HTLV tax gene relative to the human housekeeping gene RPP30. Since RPP30 is present in two copies per cell, the estimated number of cells is calculated by dividing the RPP30 copy number by two. The PVL is then calculated as:

PVL (%) = [HTLV tax copies / (RPP30 copies / 2)] × 100. The PVL was then subsequently normalized to the number of lymphocytes in the sample, as determined by flow cytometry. The resulting lymphocyte-normalized PVL values were reported by the laboratory.

### Statistical analysis

Analyses were primarily descriptive, Continuous variables were summarized using means (min/max) and standard deviation (SD) or medians and interquartile ranges (IQRs), while categorical variables were presented as frequencies and percentages. Comparisons of continuous variables were performed using nonparametric tests, specifically the Mann–Whitney U test for two-group comparisons and the Kruskal–Walli’s test for multiple groups and post hoc analyses with Dunn’s multiple comparison test when appropriate. Categorical variables were compared using the Chi-square test or Fisher’s exact test, as appropriate. Correlations between continuous variables were assessed using Spearman’s rank correlation coefficient. Analyses were performed using available case data, without imputation of missing values. All *p*-values were two-sided, and statistical significance was set at *p* < 0.05. Data were analyzed using GraphPad Prism (Version 10.1.2).

## Results

### Cohort characteristics

Between January 2013 and May 31, 2024, 102 individuals with HTLV infection referred to the Infectious Diseases outpatient clinic at Karolinska University Hospital, Huddinge, Stockholm, Sweden, were included in the study. All patients attending the clinic had been tested positive for HTLV-1 or HTLV-2 prior to the referral. The cohort characteristics are presented in Table 1.

**Table 1:** Characteristics of the HTLV cohort

Characteristic	Female	Male	Total
**Overall cohort**			
Patients, n (%)	55 (54%)	47 (46%)	102 (100%)
Age, years, median (IQR; range)	56 (44–68; 32–81)	56 (40–63; 23–78)	56 (42–64; 23–81)
Age at diagnosis, years, median (IQR; range)	46 (33–56; 11–​72)	42 (31–55; 11–71)	45 (32–56;​11–72)
Years since diagnosis, median (IQR; range)	10.0 (4–16; 0–28)	9.3 (1–16;0–27)	9.7 (4–15;0–28)
**HTLV-1**			
Patients, n/N (%)	47/84 (56%)	37/84 (44%)	84/102 (82%)
HAM/TSP, n/N (%; 95% CI)	7/47 (14.9%; 7.4–27.7)	3/37 (8.1%; 2.8–21.3)	10/84 (11.9%; 6.6–20.6)
ATLL, n/N (%; 95% CI)	2/47 (4.3%; 1.2–14.3)	0/37 (0%)	2/84 (2.4%; 0.7–8.3)
HIV coinfection, n/N (%; 95% CI)	1/47 (2.1%; 0.4–11.1)	1/37 (2.7%; 0.5–13.8)	2/84 (2.4%; 0.7–8.3)
**HTLV-2**			
Patients, n/N (%)	10/18 (56%)	8/18 (44%)	18/102 (18%)
HIV coinfection, n/N (%; 95% CI)	3/18 (30%;10.8–60.3)	8/8(100%;67.6–100)	11/102 (10,8%;6,1–18,3)
**Presumed mother-to-child transmission**			
MTCT in Sweden, n/N (%)	2/55 (3.6%)	2/47 (4.3%)	4/102 (3.9%)
MTCT outside Sweden, n/N (%)	2/55 (3.6%)	3/47 (6.4%)	5/102 (4.9%)
**Comorbidities**			
Autoimmune disease†, n (%)	6 (10.9%)	0 (0%)	6 (5.9%)
Arthralgia/back pain, n (%)	17 (30.9%)	7 (14.9%)	24 (23.5%)
Intravenous drug use, n (%)	5 (9.1%)	13 (27.7%)	18 (17.6%)
Psychiatric disease‡, n (%)	2 (3.6%)	3 (6.4%)	5 (4.9%)
Strongyloidiasis, n (%)	2 (3.6%)	3 (6.4%)	5 (4.9%)

† Autoimmune diseases include hypothyroidism, rheumatoid arthritis, and vitiligo

‡ Psychiatric diseases include schizophrenia and anxiety

Abbreviations: ATLL, adult T-cell leukemia/lymphoma; CI, confidence interval; HAM/TSP, HTLV-1-associated myelopathy/tropical spastic paraparesis; HIV, human immunodeficiency virus; HTLV, human T-cell lymphotropic virus; IQR, interquartile range; MTCT, mother-to-child transmission

The median age of the cohort was 56 years (IQR 42–62; range 23–81) and 54% were women. The majority (82%) were HTLV-1 positive, while 18% were HTLV-2 positive. Co-infection with HTLV-1 and HIV was low in our cohort (2%), while co-infection with HTLV-2 and HIV was observed in 11% of cases—all with a history of intravenous drug use (IDU).

Notably, four cases of mother-to-child transmission (MTCT), were identified among patients born in Sweden to HTLV-1 positive mothers who were unaware of their HTLV-status during pregnancy and breastfeeding. Additional five cases of MTCT were also present among patients from high-endemic regions including South America and Middle East. Ten of the 84 HTLV-1-infected individuals (12%) were diagnosed with HTLV-1-associated myelopathy/tropical spastic paraparesis (HAM/TSP), of those 70% were women. The majority of HAM/TSP patients originated from Middle East and South America. Sexual transmission was the most common mode of transmission among this group (seven cases), followed by MTCT (two cases), while the transmission route was unknown in one case. The mean age of HAM/TSP patients was 58 years (SD 18; 95%Cl 45–71), and the average age at HAM/TSP diagnosis was 45 years.

Two patients, both older adults (>60 years), were diagnosed with ATLL. In both, the disease followed an aggressive and non-curable course despite treatment with Zidovudine and pegylated interferon-alpha.

### Reason for testing

**Fig. 1:**
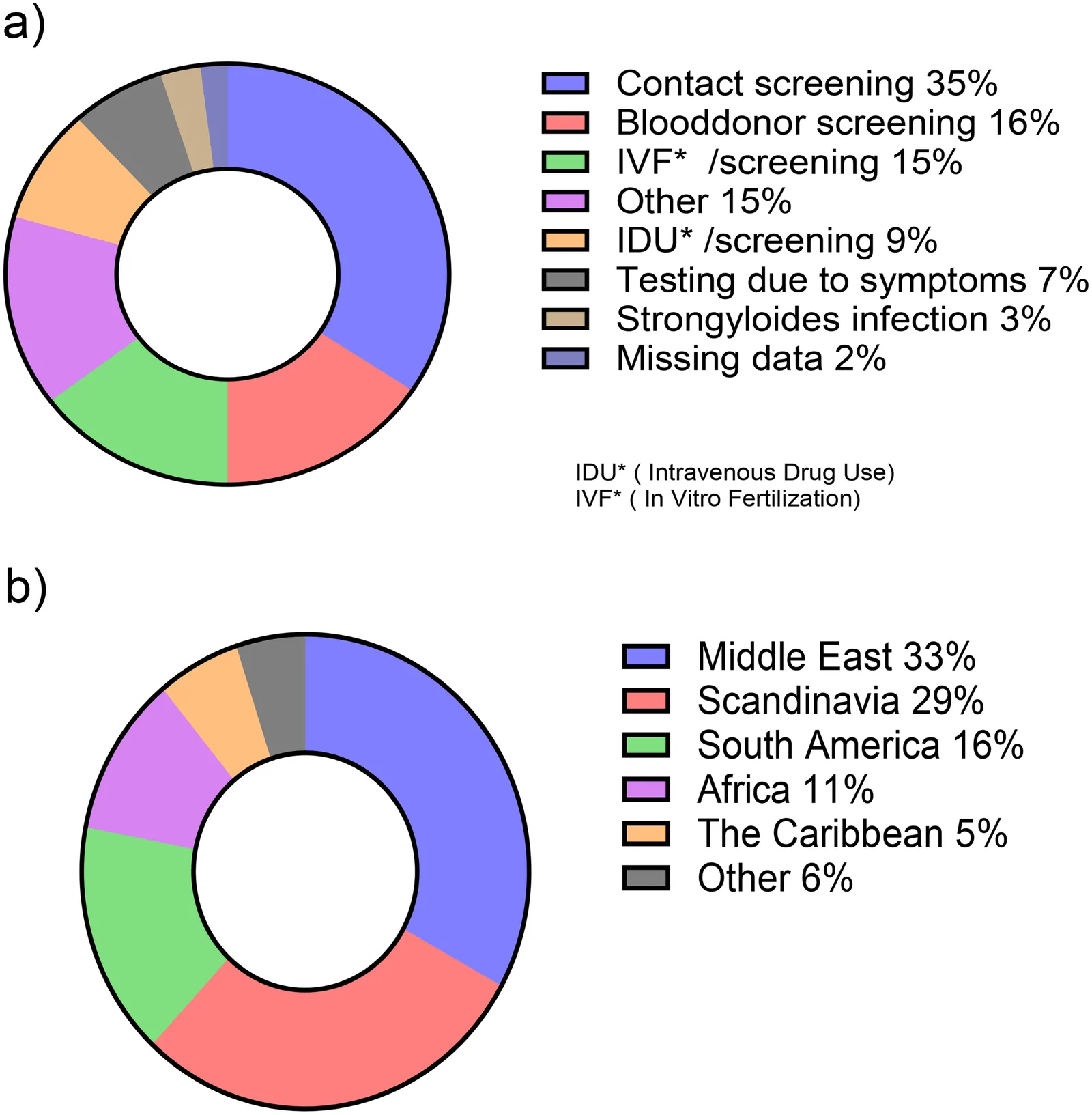
Reason for testing (**a**) and region of origin (**b**) In HTLV cohort followed at Karolinska University Hospital. **a**) Reasons for HTLV testing prior to referral. Distribution of reasons for HTLV testing among individuals with confirmed HTLV infection prior to referral to our center. Contact screening was the most common reason for testing 35%; *n* = 35, followed by blood donor screening 16%; *n* = 16, IVF screening 15%; *n* = 15, other reasons 15%; *n* = 15, intravenous drug use (IDU) screening 9%; *n* = 9, testing prompted by symptoms 7%; *n* = 7 and testing for Strongyloides infection 3%; *n* = 3. The reason for testing was missing for 2%; *n* = 2 of individuals. **b**) Geographic region of birth among individuals with HTLV infection. Participants were categorized into geographic regions according to their country of birth. The Middle East accounted for 33%; *n* = 33, Scandinavia 29%; *n* = 29 South America 16%; n16, Africa 11%; *n* = 11 the Caribbean 5%; *n* = 5, and other regions 6%; *n* = 6

The most common reason for HTLV testing was contact tracing (35%), followed by blood donor screening (16%) and in vitro fertilization (IVF) screening (15%), reflecting routine HTLV screening practices in Sweden as seen in Fig. 1a.

Only seven individuals were diagnosed due to symptomatic disease presentation-six patients presented with neurological symptoms and one with adult T-cell leukemia/lymphoma (ATLL).

### Region of origin

The majority of patients originated from the Middle East (33%), primarily Iran, followed by Scandinavia (29%), South America (16%, mainly Peru), Africa (11%), and the Caribbean (5%) as presented in Fig. 1 b.

### PVL

**Fig. 2:**
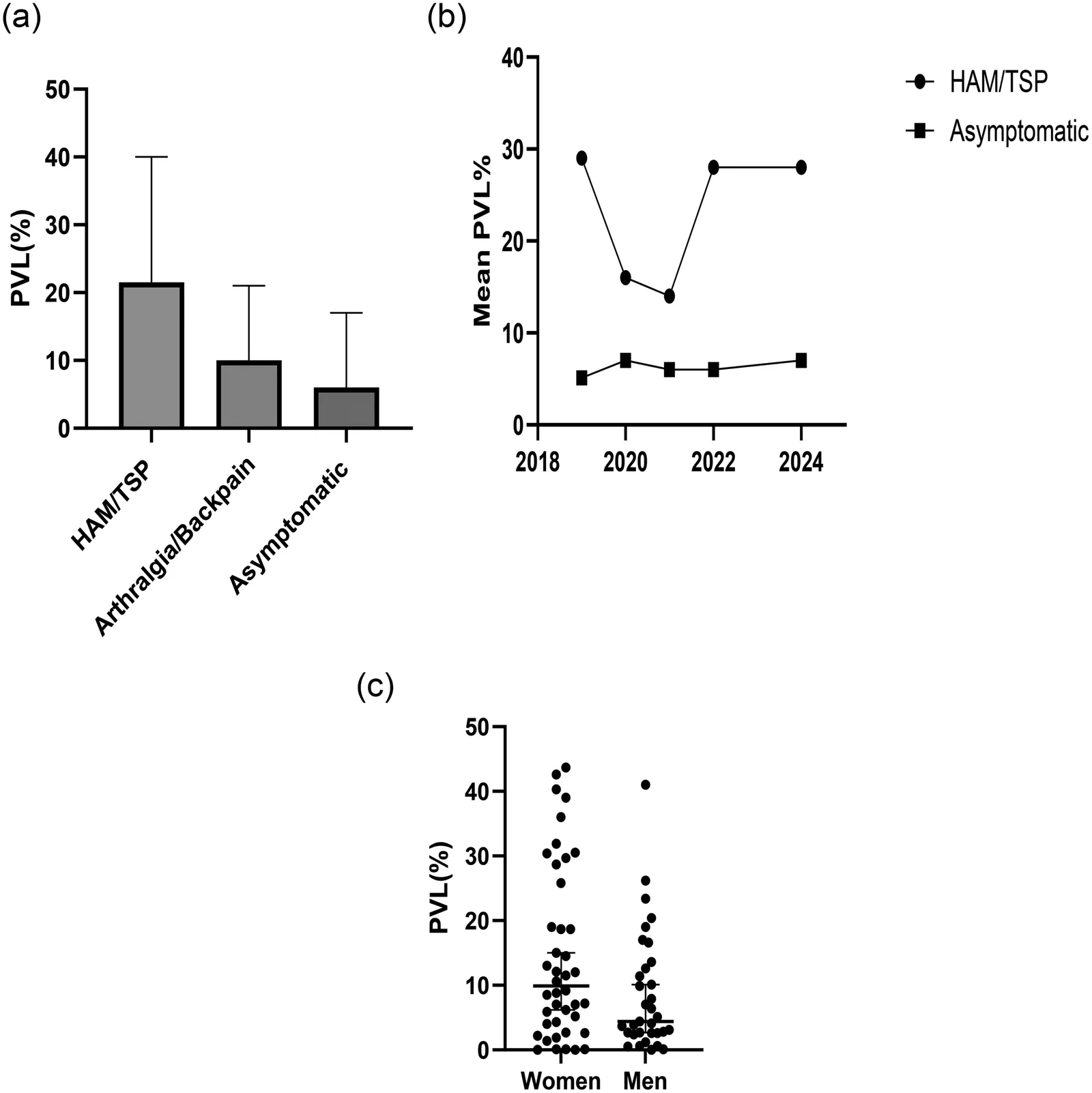
HTLV-1 proviral load (PVL) in the cohort **a**) Baseline PVL in individuals with and without symptoms, **b**) Kinetics in PVL during follow up **c**) Baseline PVL in women and men​. **a** Baseline proviral load (PVL) according to clinical presentation. Data are presented as median and interquartile range (IQR). HAM/TSP, *n* = 10; arthralgia/back pain, *n* = 13; asymptomatic, *n* = 53. Differences between groups were assessed using the Kruskal–Wallis test (*p* = 0.058). Missing values were excluded from the analysisn. **b** Mean PVL (%) by year in individuals with HAM/TSP and asymptomatic HTLV-1 infection, 2019–2024. Means were calculated using available observations for each year; n per year asymptomatic = 31,17,14,14,17. n per year HAM/TSP = 5,6,6,5,4. **c** Baseline HTLV-1 proviral load according to sex. Baseline HTLV-1 proviral load (PVL) in women (*n* = 42) and men (*n* = 33). Individual observations are shown with median and interquartile range (IQR). Groups were compared using Mann–Whitney U test (*p* = 0.08)

At baseline, proviral HTLV load (PVL) differed numerically across the clinical groups, with the highest median PVL observed in patients with HAM/TSP (21.5%; IQR 5.75–40%), followed by patients with arthralgia/back pain (10%; IQR 3.5–21%) and asymptomatic carriers (6%; IQR 2.5–17%). However, the overall difference between the groups did not reach statistical significance as presented in Fig. 2 a. (Kruskal Wallis test *p* = 0.058).

Longitudinally, PVL kinetics differed between patients with HAM/TSP and asymptomatic HTLV carriers as seen in Fig. 2b.

Baseline PVL did not differ significantly between women and men as presented in Fig. 2c. (Mann–Whitney U test, *p* = 0.08).

### Immunology

**Fig. 3:**
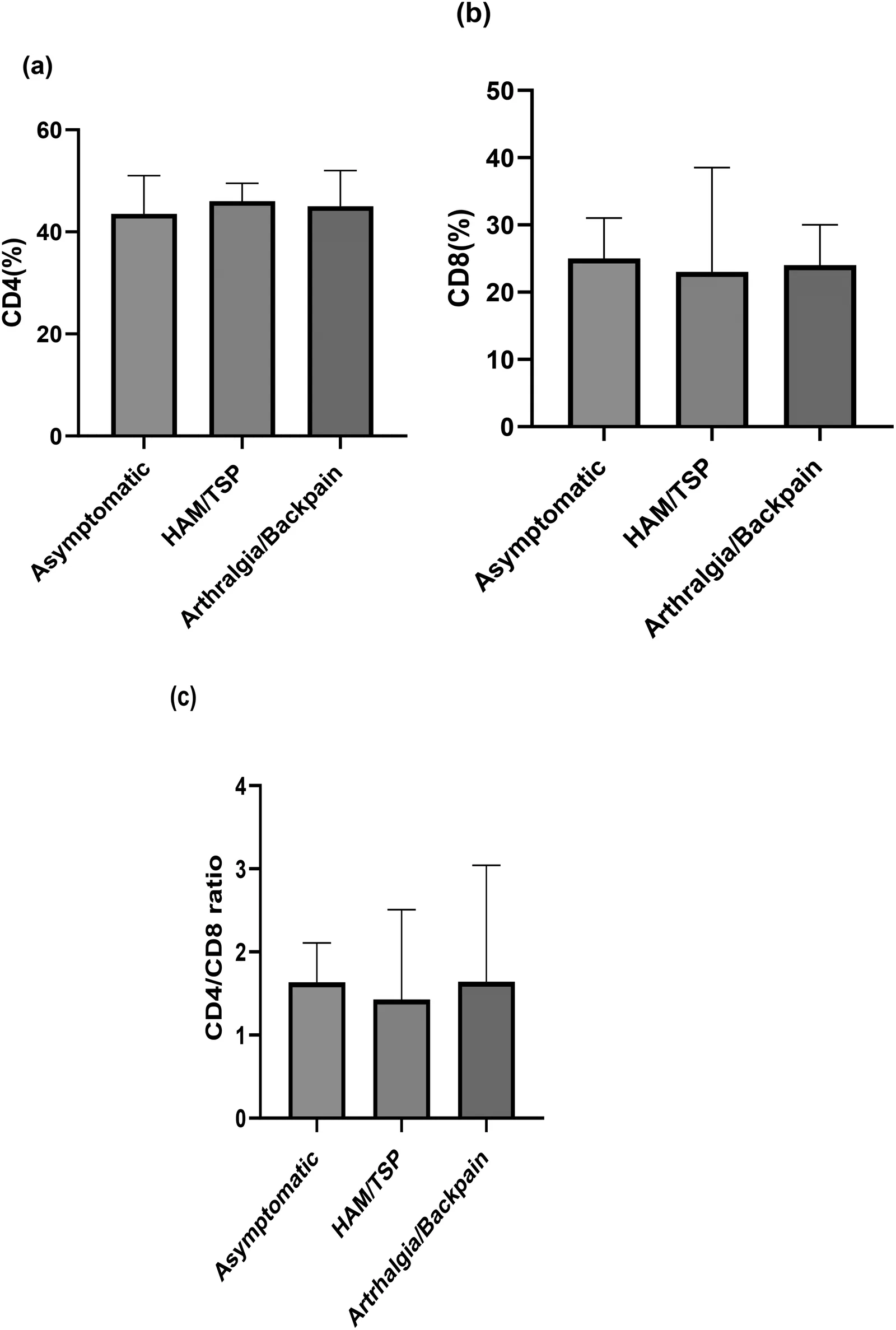
Distribution of CD4 (**a**) and CD8 T cells (**b**) and CD4 /CD8 ration (**c**) in patients with HTLV-1 infection, stratified by clinical disease manifestations. **a** CD4 levels according to clinical presentation. CD4 levels are presented as median with interquartile range (IQR). Comparison between groups were assessed using the Kruska Wallis test (*p* = 0.74) Asymptomatic, *n* = 46; HAM/TSP, *n* = 9; arthralgia/back pain, *n* = 13. **b** CD 8 levels according to clinical presentation. Data are presented as median and interquartile range (IQR). Differences between groups were assessed using the Kruskal–Wallis test (*p* = 0.99). Asymptomatic *n* = 46; HAM/TSP *n* = 9; Arthralgia/backpain; *n* = 13. **c** CD4:CD8 ratio according to clinical presentation. Data are presented as median and interquartile range (IQR). Asymptomatic, *n* = 22; HAM/TSP, *n* = 9; Arthralgia/Back pain, *n* = 6. Differences between groups were assessed using the Kruskal–Wallis test (*p* = 0.78)

Moreover, we found no significant differences in the distribution of CD4^+^ or CD8^+^ T-cell frequencies presented in Fig. 3a and b, nor in the CD4/CD8 ratio, when comparing individuals with HAM/TSP versus asymptomatic infection and those with arthralgia/back pain presented in Fig. 3c.

Regulatory T cell proportions differed significantly across the three subgroups (Kruska Wallis *p* = 0.048). Patients with HAM/TSP (median 18%, IQR 11,85–31,85%) and Arthralgia/Backpain (median 19%, IQR 11–39%) had higher T-reg proportions compared to asymptomatic individuals (median 10%, IQR 8,1–14,25%). However post hoc analysis with Dunn’s multiple comparison test did not reveal any statistically significant pairwise difference after adjustment for multiple comparisons (all adjusted *p* > 0.05).

**Fig. 4:**
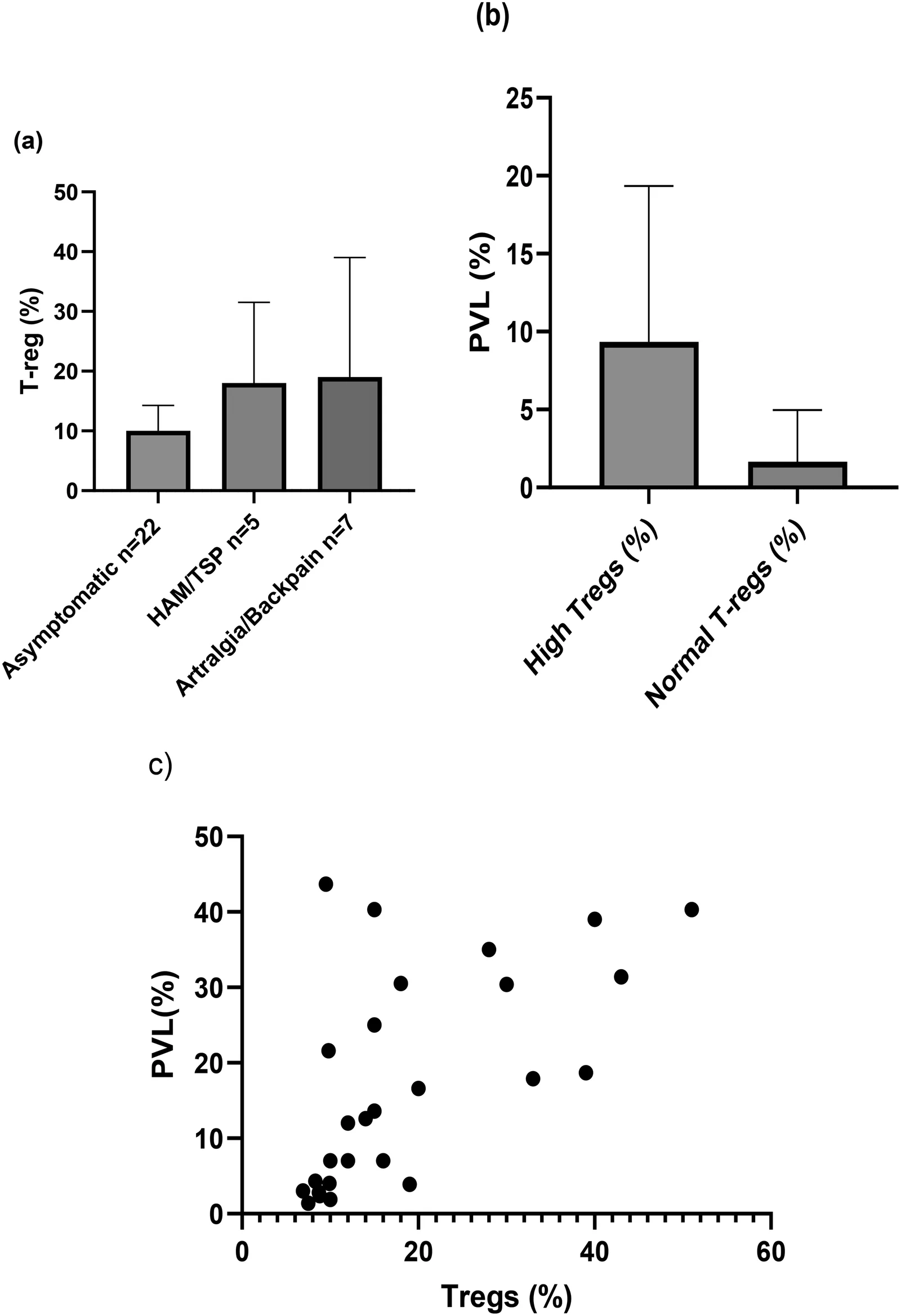
Association of regulatory T cells (tregs) with clinical presentation and proviral load (PVL) in patients living with HTLV-1. Distribution of T regs (**a**); PVL in patients with high vs normal T regs (**b**) and spearman correlation between PVL and T regs (**c**). **a** Regulatory T-cell distribution in the cohort in asymptomatic individuals (*n* = 22), patients with HAM/TSP (*n* = 5), and patients with arthralgia/backpain (*n* = 7). Data are presented as median and interquartile range (IQR). Differences between groups were assessed using the Kruskal–Wallis test (*p* = 0.049), followed by Dunn’s multiple comparisons test. No statistically significant differences were observed in pairwise comparisons after adjustment for multiple testing, all adjusted *p*=>0.05. **b** PVL (%) according to regulatory T-cell frequency. HTLV-1 proviral load (PVL) in individuals with Treg frequencies within the normal range (*n* = 10) and elevated Treg frequencies (*n* = 38). Data is presented as individual observations with median and interquartile range (IQR). Groups were compared using Mann–Whitney U test (*p* = 0.0066). Data is presented as median and interquartile range (IQR). Mann-Whitney test was performed to access correlation between PVL and high T-regs (%) *n* = 10 and Normal T-regs (%) *n* = 38 (*p* = 0.006). **c** Correlation between regulatory T-regs (%) and PVL (%). Scatter plot of T-reg (%) and HTLV-1 proviral load (PVL, %) among HTLV-1-infected individuals with available measurements. Each point represents one participant. Spearman rank correlation analysis demonstrated a positive correlation between Treg frequency and P​VL (*r* = 0.64, *p* = 0.0003)

In addition, as seen in Fig. 4b, patients with higher PVL exhibited increased Treg frequencies, suggesting a potential association between Treg expansion and proviral burden. Indeed, a strong positive correlation was observed between PVL and Treg percentages (*r* = 0.64, *p* = 0.0003) presented in Fig. 4c.

## Discussion

Here, we have described the clinical and immunological characteristics of 102 individuals with HTLV infection followed at Karolinska University Hospital, representing to our knowledge, the largest reported HTLV cohort in the Nordic countries. This study provides insights into the demographic and clinical characteristics, diagnostic pathways and immune profiles of individuals with HTLV infection managed at a tertiary referral center in a low-prevalence setting.

Most cases in our cohort were HTLV-1 infections. This is consistent with global epidemiology and reflects our patient population, in which a substantial proportion of individuals were born in HTLV-endemic regions, including the Middle East and South America. Studies from other low prevalent European countries have similarly reported a higher HTLV-1 prevalence among individuals originating from endemic regions. For example, a study from England showed a higher prevalence of HTLV-1 in women originating from the Caribbean (1,4%) compared to women born in England (0,03%) [5]. Similarly, in Spain the overall seroprevalence among pregnant women is below 0,02% but in pregnant women from Latin America and Africa it was 0,2% [13]. Notably, 29% of individuals in our cohort were born in Scandinavia, indicating that HTLV infection is not restricted to individuals born in HTLV-endemic regions.

Awareness of HTLV infection in Sweden has been reported to be limited, both among the general population and healthcare professionals [6]. Although, the present study was not designed to asses awareness, testing frequency or the extent of underdiagnosed HTLV infection in Sweden, limited awareness likely contributes to low testing frequency, delayed diagnosis, and continued silent transmission of HTLV infection as supported by data from a previous study in Spain [14]. Furthermore, HTLV infection is often perceived as a clinically insignificant or asymptomatic condition, which may reduce clinical focus and surveillance. However, recent data challenge this perception: a systematic review and metanalysis of epidemiological studies has demonstrated that HTLV-1 infection is associated with a 57% increased risk of premature mortality, independent of ATLL and HAM/TSP [15]. Thus, increased awareness and recognition of HTLV-1 infection are essential, for not only early identification and management of symptomatic cases, but also to guide appropriate monitoring, prevention, and long-term care strategies for asymptomatic carriers [16].

In our cohort, the majority of HTLV diagnoses resulted from routine or contact-based screening, with only 7% detected through symptoms, underscoring that most infections are asymptomatic at diagnosis. In Sweden, HTLV screening has been implemented for first-time blood donors since 1994, and routine HTLV testing is performed prior to IVF treatment; however, HTLV is not currently included in the national antenatal screening program. Mother-to-child transmission is a well-established route of HTLV-1 transmission and has been associated with an increased lifetime risk of HTLV-related disease, particularly ATLL, which carries high mortality [17]. In Japan, a high -prevalence country, national antenatal HTLV screening was introduced in 2011 together with recommendations aimed at reducing MTCT, resulting in a substantial decline in new HTLV infections [18]. Breastfeeding is a major route of MTCT, and avoidance or limitation of breastfeeding has been implemented as an important preventive strategy [19, 20].

In four individuals born in Sweden in our cohort, MTCT was considered a probably route of infection based on the available clinical and epidemiological information; however, the retrospective study design did not allow the route of transmission to be confirmed. These observations should therefore be interpreted cautiously and cannot be used to estimate the frequency of MTCT or the prevalence of HTLV in Sweden. Population-based studies are needed to better characterize HTLV epidemiology in Sweden and to evaluate the potential role and appropriate targeting of antenatal screening strategies in this low-prevalence setting [21]. Clinically, 12% of HTLV-1–infected individuals in our cohort were diagnosed with HAM/TSP, with a predominance of female patients. Our findings are consistent with previous studies showing than females face a higher risk to develop HAM/TSP than men [22–24]. Patients with HAM/TSP had the highest median PVL, while elevated PVL was also observed among individuals reporting arthralgia and back pain. Although these findings did not reach statistical significance, they may suggest a broader spectrum of HTLV-associated inflammatory manifestations that are not captured by current diagnostic frameworks [25]. Additionally, even if the pathophysiology of HAM/TSP remains incompletely understood and effective treatments are lacking, the association between high PVL and increased HAM/TSP risk has been demonstrated in several studies [24, 26, 27]. However, PVL alone is not a reliable predictor of disease progression, as many asymptomatic HTLV-1 carriers with high PVL never develop HAM/TSP [26, 28].

A distinct viral–immune phenotype associated with elevated HAM/TSP risk has been proposed [26], and the clinical transition to HAM/TSP is typically gradual, making early detection challenging. In addition to neurological manifestations required for HAM/TSP diagnosis, previous studies have reported other HTLV-related clinical symptoms [29]. Further longitudinal studies are needed to determine whether clinical, virological, or immunological characteristics may help identify individuals at increased risk of developing HTLV-1-associated disease.

Immunological profiling in our cohort revealed no significant differences in CD4+ or CD8+ T-cell percentages, nor in the CD4/CD8 ratio, between the groups. Although regulatory T cell proportions were numerically higher in both the HAM/TSP and arthralgia/back pain groups than in asymptomatic individuals post hoc analyzes did not reach statistical significance. The small symptomatic subgroups may have limited our ability to detect differences. The positive correlation between PVL and Treg frequency further supports the hypothesis that immune dysregulation, particularly through Treg expansion—may play a key role in HTLV pathogenesis [30]. As reviewed by Shafiei et al., HTLV-1 preferentially infects Tregs, leading to numerically increased but functionally impaired Tregs cell population. This dysfunction is partly mediated by viral proteins like Tax, causing reduced expression of FoxP3, TGF‑β, and IL-10. Additionally, infected cells exhibit marked plasticity, with frequent conversion to Th1- and Th17-like phenotypes. This contributes to a pro-inflammatory environment, particularly in the CNS in HAM/TSP [31] and may represent a potential target for future immunomodulatory therapy in HTLV-associated autoimmune complications. However, given the small sample size and cross-sectional nature of the immunological analyses, our findings should be regarded as exploratory and do not establish a role for Treg alterations in the pathogenesis of HAM/TSP or other clinical manifestations. Longitudinal studies in larger cohorts are needed to further investigate the relationships between PVL, regulatory T-cell alterations, and clinical outcomes.

Several limitations should be considered when interpreting our findings. First, the retrospective, descriptive, single-centre design and recruitment from a tertiary referral clinic limit generalizability to the broader population of people living with HTLV in Sweden. The geographic distribution of our cohort therefore cannot be used to infer national HTLV prevalence or temporal trends. Referral patterns may also favour individuals with symptomatic or clinically recognized disease, potentially leading to an overrepresentation of HAM/TSP and ATLL. In addition, the cross-sectional immunological profiling reflects a single time point and therefore cannot capture changes in HTLV-related immunopathology over time. The small sample size in the clinical subgroups limits statistical power, particularly for comparison of PVL and T-reg proportions, and these findings should therefore be interpreted as exploratory. Finally, although this is the largest HTLV cohort reported in Sweden to date, its relatively small-size limits statistical power to detect subtle associations and characterize rare clinical complications.

In conclusion, our findings provide valuable insights into the clinical, virological, and immunological characteristics of individuals with HTLV infection followed at a tertiary referral center in Sweden. The findings add knowledge to the limited data on HTLV-infection in low prevalence European settings and identify observations that warrant further investigations.

## Data Availability

Data is not publicly available but may be shared upon reasonable requests for scientific collaboration.
